# Identification of Genomic Regions Associated with Peanut Rust Resistance by Genome-Wide Association Studies

**DOI:** 10.3390/plants14081219

**Published:** 2025-04-16

**Authors:** Xinlong Shi, Ziqi Sun, Feiyan Qi, Suoyi Han, Yixiong Zheng, Wenzhao Dong, Maoning Zhang, Xinyou Zhang

**Affiliations:** 1 The Shennong Laboratory/Institute of Crops Molecular Breeding, Henan Academy of Agricultural Science, Zhengzhou 450002, China; shixinlong87@163.com (X.S.); sunziqi777@163.com (Z.S.); qifeiyan85@163.com (F.Q.); suoyi_han@126.com (S.H.); dongwenzhao@126.com (W.D.); zhangmaoning@gs.zzu.edu.cn (M.Z.); 2 College of Agriculture/Tree Peony, Henan University of Science and Technology, Luoyang 471023, China; 3 College of Agriculture, Zhongkai University of Agriculture and Engineering, Guangzhou 510225, China; gdsscqs@163.com

**Keywords:** peanut, peanut rust resistance, GWAS, SNPs, candidate gene

## Abstract

Peanut rust, caused by *Puccinia arachidis* Speg., is one of the most significant leaf diseases globally, and has a severe impact on peanut yield and quality. The development of disease-resistant varieties is recognized as an effective strategy to mitigate the damage caused by peanut rust. However, the research foundation for understanding peanut rust remains relatively limited. In this study, we identified significant single nucleotide polymorphisms (SNPs) associated with peanut rust resistance using a natural population consisting of 353 peanut germplasm accessions. These accessions were analyzed based on resequencing data and rust disease phenotypes across one laboratory test and three field trials. A total of 18 significant SNPs were identified on chromosomes A05 (5 SNPs), A08 (7 SNPs), and A12 (6 SNPs). Notably, three SNPs—Arahy.05_93085395, Arahy.05_93114354, and Arahy.12_4097252—were consistently detected across multiple environments. Within their confidence intervals, 48 genes were annotated, including 9 NLR domain-containing genes functionally related to plant disease resistance, which may serve as candidate genes for peanut rust resistance. This study provides insights into the regulatory mechanisms underlying peanut rust resistance.

## 1. Introduction

The cultivated peanut (*Arachis hypogaea* L.) is one of the most economically important oilseed crops [1,2] worldwide. Peanuts are cultivated across all continents, with Asia being the largest producer [3,4]. Peanut seeds are rich in protein and lipids, making them a globally recognized healthy food [4]. In recent years, the demand for peanut oil and related by-products has continued to rise. Despite an increase in peanut planting areas, challenges in yield and quality remain significant. Additionally, various biotic and abiotic stresses occur frequently, particularly fungal, bacterial, and viral diseases, which have become increasingly severe (China National Bureau of Statistics, https://www.stats.gov.cn/, accessed on 12 May 2024). Therefore, it is imperative to intensify research on disease resistance to ensure high yields, superior quality, and the sustainable development of the peanut industry.

Peanut rust, caused by the fungus *Puccinia arachidis* Speg., is a worldwide leaf disease that seriously affects peanut yield and quality [5,6,7]. Peanut rust can lead to huge yield losses and also cause deterioration in appearance quality, such as peanut pod and seed kernel uniformity. Moreover, it can reduce nutritional quality, affecting oil content and oil components [7,8,9]. Therefore, a lot of research work is needed to reduce the occurrence of rust disease and ensure the safety of oil production [9]. The current strategies used to control peanut rust mainly include cultivation patterns, chemical control, and resistance varieties [10,11,12]. Improving cultivation patterns and the application of chemicals can control the occurrence of rust to some extent, but these methods increase production costs and pose risks to food safety and the environment. Breeding resistant varieties represents the most effective and economical way to prevent peanut rust [11].

In recent years, a large number of studies on peanut rust resistance breeding have been carried out domestically and internationally, and many rust-resistant germplasms have been discovered, such as Tarapoto [9], ICGV87354 [12], Fuhua 6 [13], and Shanyou 27 [14]. However, these resistant germplasms are linked to adverse agronomic traits and have limited application in production due to their lower yield potential. Therefore, new rust-resistant peanut varieties are critically needed in production. Currently, the rust-resistant varieties widely planted in production are mainly selected through traditional hybrid breeding techniques, which are time-consuming and labor-intensive. Compared with traditional breeding methods, molecular marker-assisted selection can greatly improve the accuracy of selection in hybrid progeny, reduce breeding costs, and increase breeding efficiency; it has been widely applied in rust resistance breeding for *Coffea arabica* L. [15], *Triticum aestivum* L. [16], *Glycine max* L. [17], *Zea mays* L. [18], and *Panicum miliaceum* L. [19].

Although some progress has been made in cultivating rust-resistant varieties [20,21,22], the genetic research on peanut rust resistance is still in its infancy. Khedikar et al. [23] detected 12 QTLs related to rust resistance through SSR markers; Pandey et al. [24] discovered 25 candidate genes related to rust on chromosome A03 through QTL-seq; Sujay et al. [25] developed three rust-resistant SNP markers using conventional QTL mapping methods in susceptible populations, rust-resistant parents of RIL populations, and 11 introgression lines. The genetic research on peanut rust is relatively weak and there is still a lack of new loci related to regulating peanut rust resistance [26]. Therefore, it is of great significance to develop peanut rust resistance breeding to analyze genetic mechanisms, excavate new loci, predict candidate genes, and develop new molecular markers.

Genome-wide association analysis (GWAS) represents an efficient and widely adopted genetic research strategy that has been instrumental in localizing and mining important traits in peanuts [27,28,29,30]. In this study, we focus on a natural population comprising 353 peanut germplasm resources, systematically evaluating their rust disease phenotypes under diverse field and laboratory conditions. Then, GWAS analysis was performed using resequencing data obtained from our research team [29], with the aim of identifying resistance loci associated with peanut rust disease and predicting candidate genes linked to rust resistance. This research provides a solid foundation for the genetic improvement of peanut rust disease resistance.

## 2. Results

### 2.1. Resistance Evaluation for Peanut Rust

**Figure 1:**
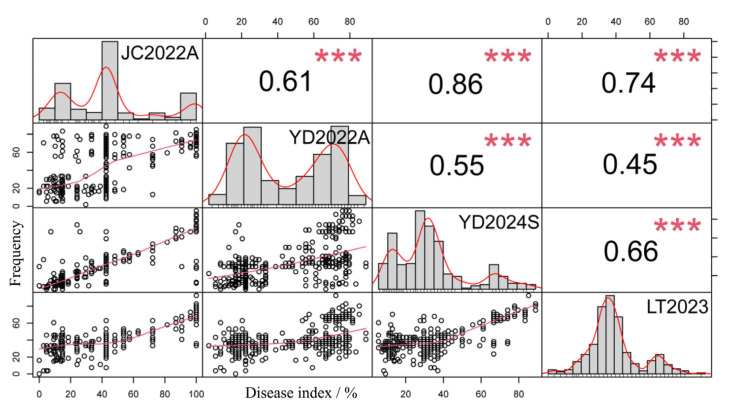
Phenotypic distribution and correlation of PRDI in natural populations in 4 environments. Note: JC2022A, planted in Jiangcheng District, Yangjiang City, Guangdong Province, China in the autumn of 2022; YD2022A, planted in Yingde City, Qingyuan City, Guangdong Province, China in the autumn of 2022; YD2024S, planted in Yingde City, Qingyuan City, Guangdong Province, China in the spring of 2024; LT2023, planted in Shennong Laboratory, Pingyuan New District, Xinxiang City, Henan Province, China in 2023. *** Indicated an extremely significant correlation at the 0.001 level (*p* < 0.001).

In this study, the rust resistance of 353 peanut varieties was evaluated through laboratory tests (LT2023) and field trials (JC2022A, YD2022A, YD2024S) from 2022 to 2024 (Table 1 and Figure 1). It was found that the mean value of the peanut rust condition index (PRDI) in four environments ranged from 33.94% to 44.90%, and the coefficient of variation was 37.92% to 63.52% (Table 1 and Supplementary Table S1), indicating that the population had rich phenotypic variation, which was conducive to the mining of the genetic locus of peanut rust. Except for the YD2022A environment, where the absolute value of skewness was greater than 1, the absolute values of skewness and kurtosis in the other three environments (JC2022A, YD2024S, and LT2023) were less than 1, and the phenotype did not exactly fit to a normal distribution (Table 1 and Figure 1). The Pearson (Pearson) correlation coefficient was calculated to show a very significant correlation (*p* < 0.001) for PRDI in different years and environments (Figure 1). ANOV further showed (Table 2) that PRDI varied significantly between environments, genotypes, and the environment and genotype (*p* < 0.001), with a heritability of 0.58, indicating that peanut rust resistance is significantly affected by both the environment and genotype.

### 2.2. Genome-Wide Association Analysis of Peanut Rust Resistance

**Figure 2:**
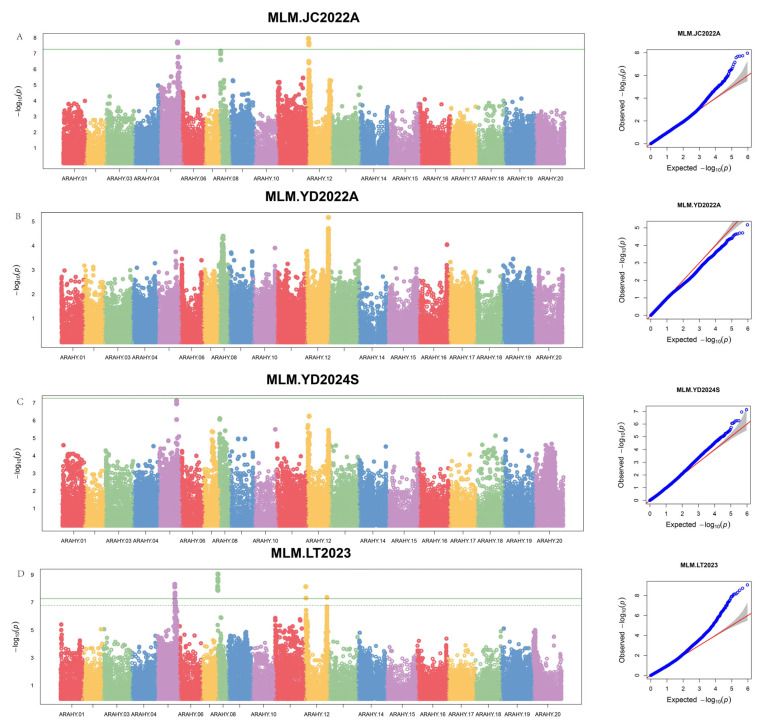
Manhattan plots and Q-Q plots for PRDI.

In this study, 935,231 high-quality loci obtained by the research group were used to conduct a genome-wide association analysis of peanut rust resistance based on the MLM model [29]. Finally, 18 significant SNP sites were identified under the threshold line-log10 (0.05/935,231) ≈ 7.27, distributed across three chromosomes, namely, A05, A08, and A12 (Figure 2 and Supplementary Table S2). Among these, three significant SNP sites were identified in multiple environments, namely, Arahy.05_93085395, Arahy.05_93114354, and Arahy.12_4097252, with −log10 (*P*) value ranges of 7.67–8.32, 7.72–8.12, and 7.95–8.14, respectively (Figure 2 and Table 3).

### 2.3. Candidate Gene Prediction

Genes within 200 kb upstream and downstream of the significant SNP were selected as confidence intervals according to the reference genome annotation database of the cultivated species peanut, Tifrunner1 [30]. A total of 282 genes were annotated in the 18 significant SNP confidence intervals (Supplementary Table S3). Among these, three SNP sites were stable in multiple environments, namely Arahy.05_93085395, Arahy.05_93114354, and Arahy.12_4097252. Both Arahy.05_93085395 and Arahy.05_93114354 are located on chromosome A05 with a close physical location, so their confidence intervals were merged into A05:92885395-93314354, with a length of 428.959 kb, and encompassing 15 genes within the annotation interval. Arahy.12_4097252 is located on chromosome A12 with a confidence interval of A12:3897252-4297252, where 33 genes were annotated within the interval (Supplementary Table S3).

**Figure 3:**
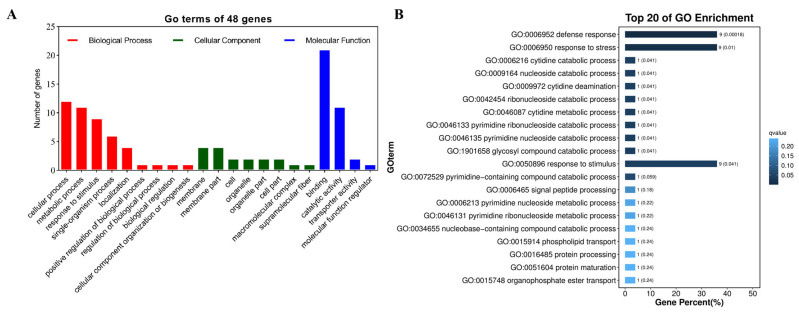
Bar graph of the GO enrichment pathway (**A**) and the top 20 of GO enrichments (**B**) for genes in candidate intervals.

Among them, 48 genes could be repeatedly annotated; these 48 genes were subjected to GO enrichment analysis, showing the top 20 significantly enriched pathways in the form of a bubble map (Figure 3 and Table 4). The results show that these genes are mainly enriched in processes related to the plant’s response to external stimuli, defense response, stress response, and other processes. Interestingly, GO: 0006952, GO: 0006950, and GO: 0050896 each had the highest count of nine genes; the functional annotation of these genes identified them as plant disease resistance genes containing the NLR domain (Table 4), which is speculated to be related to peanut rust resistance.

## 3. Discussion

Peanuts are important crops, and cultivated peanuts are divided into two subspecies, namely, the alternate flowering subspecies and the continuous flowering subspecies [31]. Krapovickas et al. [32] categorized peanuts based on whether they bloom on the main stem, dividing them into dense branch subspecies (alternate flowering subspecies) and sparse branch subspecies (continuous flowering subspecies). Within these categories, dense branch subspecies are divided into dense branch species and antler species; sparse branch subspecies are divided into pearl bean species, Peruvian species, sparse branch species, and equatorial species. In the Chinese peanut varieties and their genealogy, the alternate flowering subspecies are divided into common and dragon [33], corresponding to the branch varieties and antler species [32] in foreign classification; the continuous flowering subspecies are divided into multiple grain and pearl bean types, corresponding to pearl bean varieties and thinning varieties; moreover, there are intermediate subspecies. Zheng et al. [29] subdivided them into subspecies and subspecies and divided the 353 natural accessions in this study into 7 plant types and 12 subgroups. The identification and effective utilization of rust-resistant germplasm are key to cultivating peanut rust-resistant varieties, and the diversification of resources of cultivated peanut varieties provides great prospects for breeding programs to improve the rust resistance of peanuts [34,35].

Genome-wide association study (GWAS) analysis has led to significant advancements in uncovering the functional genome of plants and has been extensively applied to mine candidate genes for important agronomic traits [15,16,17,18,19]. GWAS relies on statistical methods to identify genetic variations associated with specific traits but also encounters certain limitations [36]. While GWAS can pinpoint loci linked to particular traits, it lacks the precision to accurately locate specific genes. Additionally, it may fail to detect rare and structural variants that could have substantial effects on phenotypes. Even when repeated loci are identified, there is a risk of false-positive and false-negative results, which complicates the screening of candidate genes [37]. Despite these challenges, GWAS findings remain highly valuable for reference. As GWAS research progresses, it is expected that existing issues will be progressively resolved, enhancing the efficiency and accuracy of gene exploration and analysis [36]. This study represents the first attempt to integrate GWAS with rust phenotypic data (peanut rust disease index, PRDI) from both laboratory tests (seedling stage) and field trials (adult stage), involving 353 peanut germplasms, to investigate the genetic basis of rust resistance. Furthermore, we identified candidate genomic regions and predicted candidate genes associated with peanut rust resistance. These findings may contribute to a deeper understanding of the induction mechanism of peanut rust resistance and provide valuable evidence for future breeding strategies targeting rust resistance.

Peanut rust is a complex quantitative trait, and relatively few studies have been conducted on the genetics of peanut rust resistance [23]. Khedikar et al. [23] identified 12 QTLs associated with rust resistance located on LG01, LG02, LG03, LG06, LG07, LG08, LG09, and LG10, explaining 1.70% to 55.20% of the phenotypic variation. Similarly, our study detected multiple SNPs across several chromosomes. Pandey et al. [24], Shirasawaet al. [38], Mondal et al. [39], and Ahmad et al. [40] all reported loci significantly associated with rust resistance on chromosome A03, In this study, GWAS analysis revealed two significant SNP sites (Arahy.05_93085395, Arahy.05_93114354) on chromosome A05. Sujay et al. [25] also identified loci on chromosome A05 linked to peanut rust resistance. Furthermore, three significant SNP sites were detected on chromosome A12 in this study. Among them, Arahy.12_4097252 was consistent with findings by Qi et al. [41] and Zhang et al. [42], who identified significant SNPs associated with bacterial wilt resistance on chromosome A12. Given that both peanut rust and bacterial wilt are bacterial biotic stresses, it is speculated that a common resistance locus may exist, suggesting that genes associated with rust resistance might reside on chromosome A12. The chromosomal location associated with rust resistance may also be influenced by the genetic background of the population and the nature of rust resistance. No other loci identified in this study have been previously reported; future investigations will utilize other populations to explore resistance loci on this chromosome.

Plant disease resistance genes (R genes) can directly or indirectly identify the effector proteins secreted by pathogens, thereby triggering disease resistance responses. Currently, most R genes in plants belong to the NBS-LRR type, which consists of three domains, namely, LRR, CC/TIR, and NB-ARC [42,43,44]. These proteins play a critical role in resisting external pathogen invasions and can identify multiple pathogens [44,45]. The NB-ARC domain is highly conserved and functions as a “molecular switch”, regulating disease resistance pathways through conformational changes upon ATP binding or ADP-mediated regulation [43]. The NBS-LRR family is large and diverse, and its member roles in disease resistance have been documented in wheat [46], soybean [47], rice [48], tobacco [49], and sunflower [50]. In peanuts, Feng et al. [51] cloned an NBS-LRR gene specifically expressed in roots, whose expression significantly increased after infection with *Aspergillus flavus*. Overexpression of the NBS-LRR gene AhRRS5 in peanuts, as demonstrated by Zhang et al. [52], enhanced resistance to tobacco mosaic virus and bacterial wilt. Zhao Xiao et al. [53] identified an NBS-LRR gene associated with peanut drought resistance, showing the highest expression in leaves. Collectively, these studies indicate that NBS-LRR genes are involved in plant stress resistance. In this study, nine NBS-LRR family genes were mined on chromosome A12 via GWAS, GO enrichment, and KEGG analysis, suggesting their potential involvement in peanut rust resistance.

The PPR family proteins, acting as trans-acting factors, play crucial roles in plant responses to abiotic and biotic stresses [54,55]. Su et al. [56] identified 179 DYW subset PPR genes in the soybean genome, demonstrating that the overexpression of GmPPR4 under salt and drought stress enhanced drought tolerance in transgenic plants. Chen et al. [57] discovered 491 PPR genes in rice through whole-genome analysis, revealing that many PPR genes were upregulated under stress conditions and potentially contributed to rice’s response to abiotic stress. Damage to PPR proteins has been shown to impair mitochondrial or chloroplast function, generating retrograde signals that regulate the expression of stress-related genes [58]. In this study, one and three PPR family genes were mined from candidate intervals on chromosomes A08 and A12, respectively, indicating their potential relevance to rust resistance. Protein kinases, enzymes catalyzing protein phosphorylation, play essential roles in plant stress responses [59,60,61]. Multiple protein kinase family genes were detected within the confidence intervals of significant SNPs, possibly participating in the peanut rust response process. Other disease resistance-related genes in the candidate interval include FkbM family methyltransferase genes [62], E3 ubiquitin-protein ligase genes [63], and transcription factors [64], warranting further exploration.

The primary objective of this study was to predict candidate genes related to peanut rust resistance, necessitating subsequent functional analyses and validations. Although numerous quantitative trait loci (QTLs) associated with rust resistance have been identified in other crops, few studies have reported genes specifically linked to peanut rust resistance, leaving the underlying genetic mechanisms largely unexplored. The single nucleotide polymorphisms (SNPs) and candidate genes identified in this study will pave the way for further cloning of functional genes involved in peanut rust resistance and for elucidating the genetic mechanisms underlying this trait.

## 4. Materials and Methods

### 4.1. Plant Materials

The materials used in this study consist of a natural population consisting of 353 peanut germplasm accessions maintained by the Institute of Crops Molecular Breeding, Henan Academy of Agricultural Science [29]. The 353 accessions were collected from 27 countries and 18 provinces of China, which included 5 peanut botanical varieties (85 var. *hypogaea*, 12 var. *hirsuta*, 26 var. *fastigiata*, 84 var. *vulgaris,* and 2 var. *peruviana*) and 2 kinds of irregular types (100 irregular-*hypogaea* types and 44 irregular-*fastigiata* types) (Supplementary Table S1) [29]. The control varieties used in the study were SK2803 (susceptive) and Zhongkaihua 826 (resistant), provided by the School of Agriculture, Zhongkai University of Agriculture and Engineering.

### 4.2. Peanut Rust Resistance Evaluation

**Figure 4:**
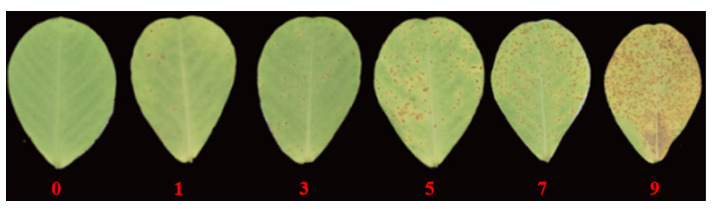
Graphical representation of indoor grading standards for peanut rust disease.

The classification standard for peanut rust disease is slightly modified according to the grade 1–9 survey standards [6], adapted by Kamble et al. [64] and formulated by ICRISAT. For indoor artificial inoculation, the disease level was determined according to the percentage of the lesion area on leaves (X), as follows: Grade 0, No spots, X = 0; Grade 1, 0 < X < 6%; Grade 3, 6% ≤ X < 25%; Grade 5, 25% ≤ X < 50%; Grade 7, 50% ≤ X < 75%; Grade 9, 75% ≤ X (Figure 4). For field identification, the disease level is classified according to the overall condition of the plant, as follows: Level 0: no symptoms; Level 1: A small number of rust spores on the lower leaves; Level 3: necrotic spots on the lower leaves, numerous spore clusters on the middle leaves, and a small number of spore clusters on the upper leaves; Level 5: prominent necrotic spots on the lower leaves, necrotic spots on the middle leaves, and a large number of spore clusters on the upper leaves; Level 7: severe damage to the lower and middle leaves, necrotic spots on the upper leaves; Level 9: severe damage to the entire plant, with leaves severely damaged or even dead. The disease index is calculated based on the disease grades and the formula for calculating the peanut rust disease index (peanut rust disease index, PRDI) is as follows [65]:

(1) $$Disease\;Index(DI)=100\times \frac{\sum (Number\;of\;diseased\;levels/Number\;of\;plants\;at\;each\;level\times representative\;value\;at\;each\;level)}{Investigate\;total\;leaf/plant\;number\times highest\;value}$$

According to the evaluation criteria of Yu et al. [66], the identified varieties were evaluated according to the disease index (DI). The resistance classification criteria were as follows: high resistance (HR) level: 0 ≤ DI < 11.11; resistant (R) level: 11.11 ≤ DI < 33.33; moderately resistant (MR) level: 33.33 ≤ DI < 55.56; susceptible (S) level: 55.56 ≤ DI < 77.78; and highly susceptible (HS) level: 77.78 ≤ DI.

### 4.3. Trial Design and Phenotypic Identification

#### 4.3.1. Field Trial Design and Management

The peanut rust resistance evaluation was conducted for 353 peanut germplasms in high-incidence areas of peanut rust. The collected fresh rust spores were poured into sterile water, then 0.1% Tween-20 (*v*/*v*) was fully mixed to make a spore suspension at a concentration of 4.0 × 10^5^ spores·mL^−1^ [65]. In the autumn of 2022, two locations in Guangdong Province were selected, namely, Jiangcheng District, Yangjiang City (JC2022A, 21°39′ N, 111°47′ E) and Yingde, Qingyuan City (YD2022A, 23°50′ N, 112°45′ E), where peanut rust naturally occurred in JC2022A and rust spores were sprayed on the entire plants at the flowering stage in YD2022A. Artificial inoculations were conducted at the flowering stage in Yingde, Qingyuan City, in the spring of 2024 (YD2024S). For each environment, a completely randomized block with two replications was designed. Ten seeds of each material were planted in two rows, with a plant spacing of 20 cm and a row spacing of 30 cm. For every 20 materials, one resistant control (Zhongkai 826) and one susceptible control (SK2803) were planted. No pesticides were sprinkled during the whole growth period, and artificial weeding and water and fertilizer measures were taken according to routine field management [67].

#### 4.3.2. Indoor Trial Design and Management

The 353 peanut germplasms were planted in the artificial climate room (LT2023) in the autumn of 2023. For each accession, nine plants were planted in three incubators (18 cm diameter, 17 cm high), with each incubator serving as a separate replicate. The artificial climate chamber was set at 25 °C, 60% humidity, and 16/8 of light/darkness. At the 6-leaf stage, the 3rd leaf from the top was labeled and inoculated by spraying spore suspension (4.0 × 10^5^ mL^−1^) + 0.1% Tween 20 [65]. The spore suspension was applied to the peanut plants by the whole-plant spray method. Inoculated leaves were evaluated 14 days post-inoculation (dpi) using an Epson Perfection V850 Pro, scanner (Hangzhou Wanshen Testing Technology, Hangzhou, China). The spot area of each diseased leaf (X) was calculated using the Leaf Area Meter Software (v.2.3.0.3; Wanshen LA-S, Shanghai, China) [68].

### 4.4. Data Analysis

Data description and statistical analysis were performed using Microsoft Excel 2010, SPSS 20.0, Origin 2018, GraphPad Prism 9.5, and R package 4.2.1 [30,69,70]. Heritability was calculated using the AOV module implemented in QTL IciMapping software [70].

### 4.5. Genome-Wide Association Study

Whole-genome resequencing (WGR) was performed on 353 accessions, resulting in 864,179 SNPs and 71,052 InDels being obtained after quality control [29,30]. The mixed linear model (MLM) [71] implemented in the R package GAPIT (v 3.0) [72] was used to identify significant associations, using population structure results from ADMIXTURE analysis (K), the first two principal components (PCs), and the flowering pattern as covariates. The genome-wide significance threshold for association was set as 0.05/*n* (where *n* denotes the number of markers) [29,30].

### 4.6. Gene Predictions Within the Candidate Interval

According to the GWAS results, the annotation of *Arachis hypogaea cv*. Tifrunner version 1 (https://peanutbase.org/, accessed on 24 March 2024) was used to predict the candidate genes for peanut rust resistance; the genomic regions within 200 kb upstream and downstream of the significant SNP sites were used as candidate regions [30]; and candidate genes for predicted floral rust resistance were reported based on previous studies.

## 5. Conclusions

In this study, GWAS analysis was used to detect peanut rust resistance loci by using resequencing data and rust phenotypes from 353 peanut germplasm materials. The results identified 18 significant SNPs associated with peanut rust resistance; these significant loci were distributed on A05, A08, and A12 chromosomes. Among them, 3 SNPs could be repeatedly detected across multiple indoor and field environments, namely, Arahy.05_93085395, Arahy.05_93114354, and Arahy.12_4097252, and 48 genes were annotated in the confidence interval. GO and KEGG analysis found 9 genes containing the NLR domain, functionally annotated as plant disease resistance-related genes, and likely related to peanut rust resistance.

## Figures and Tables

**Table 1:** Phenotype analysis of PRDI in natural populations in 4 environments.

Environment	Minimum	Maximum	Mid-Value	Average	Standard Error	Coefficient of Variation/%	Kurtosis	Skewness
JC2022A	0.00	100.00	42.60	43.82	1.48	63.52	−0.21	0.77
YD2022A	1.85	88.89	44.44	44.90	1.33	55.49	−1.62	0.06
YD2024S	5.56	88.89	29.63	33.94	1.03	57.22	0.37	0.97
LT2023	0.00	92.59	38.27	39.53	0.80	37.92	0.80	0.70

Note: JC2022A, planted in Jiangcheng District, Yangjiang City, Guangdong Province, China in the autumn of 2022; YD2022A, planted in Yingde City, Qingyuan City, Guangdong Province, China in the autumn of 2022; YD2024S, planted in Yingde City, Qingyuan City, Guangdong Province, China in the spring of 2024; LT2023, planted in Shennong Laboratory, Pingyuan New District, Xinxiang City, Henan Province, China in 2023.

**Table 2:** Analysis of variance (ANOVA) for PRDI.

Source	Degree of Freedom	Sum of Squares	Mean Squares	*F*-Value	*p*-Value	Heritability
Environment	3	56,939.477	18,979.826	283.386	0.000 ***	0.58
Genotype	352	1,024,795.125	2911.350	43.469	0.000 ***	
Environment × Genotype	1056	454,018.125	429.941	6.419	0.000 ***	
Error	1760	117,876.250	66.975			

Note: *** Indicated an extremely significant correlation at the 0.001 level (*p* < 0.001).

**Table 3:** SNPs significantly associated with PRDI, identified through GWAS.

SNP Marker	Chromosome	Location (bp)	Interval (bp)	Allele	−log10(*P*)
Arahy.05_93085395	A05	93,085,395	92,885,395–93,285,395	C/A	7.67–8.32
Arahy.05_93114354	A05	93,114,354	92,914,354–93,314,354	C/T	7.72–8.12
Arahy.12_4097252	A12	4,097,252	3,897,252–4,297,252	G/C	7.95–8.14

**Table 4:** Candidate genes associated with peanut rust resistance.

Number	Gene Name	Genn Location	Strand	Annotation
1	*Arahy.66HKIQ*	A12.3963819-3967481	+	LRR and NB-ARC domain disease resistance protein
2	*Arahy.NMJE7X*	A12.3973701-3977692	+	LRR and NB-ARC domain disease resistance protein
3	*Arahy.1DE133*	A12.3983522-3989366	+	LRR and NB-ARC domain disease resistance protein
4	*Arahy.QREM2V*	A12.3993406-3997397	+	LRR and NB-ARC domain disease resistance protein
5	*Arahy.CUPK9N*	A12.4015364-4017374	+	LRR and NB-ARC domain disease resistance protein
6	*Arahy.KD5YG3*	A12.4024728-4028719	+	LRR and NB-ARC domain disease resistance protein
7	*Arahy.0C19HY*	A12.4058399-4062049	−	LRR and NB-ARC domain disease resistance protein
8	*Arahy.V6I7WA*	A12.4236131-4239737	+	LRR and NB-ARC domain disease resistance protein
9	*Arahy.0EHV1A*	A12.4280257-4284123	+	Disease resistance protein (TIR-NBS-LRR class)

Note: + indicates that the gene sequence is positive; − indicates that the gene sequence is negative

## Data Availability

The original contributions presented in this study are included in the article/Supplementary Material. Further inquiries can be directed to the corresponding author(s).

## Supplementary Materials

The following supporting information can be downloaded at https://www.mdpi.com/article/10.3390/plants14081219/s1: Table S1: Features of the 353 Arachis hypogaea subjected to whole-genome resequencing, including the peanut rust disease index across different environments; Table S2: 18 SNPs significantly associated with peanut rust resistance, identified through GWAS; Table S3: 282 annotated genes associated with SNPs.
